# Preexisting chronic conditions for fatal outcome among SFTS patients: An observational Cohort Study

**DOI:** 10.1371/journal.pntd.0007434

**Published:** 2019-05-28

**Authors:** Shao-Fei Zhang, Zhen-Dong Yang, Mao-Lin Huang, Zhi-Bo Wang, Yuan-Yuan Hu, Dong Miao, Ke Dai, Juan Du, Ning Cui, Chun Yuan, Hao Li, Xiao-Kun Li, Xiao-Ai Zhang, Pan-He Zhang, Xian-Miao Mi, Qing-Bin Lu, Wei Liu

**Affiliations:** 1 State Key Laboratory of Pathogen and Biosecurity, Beijing Institute of Microbiology and Epidemiology, Beijing, PR China; 2 The 990 Hospital of Chinese People's Liberation Army Joint Logistic Support Force, Xinyang, PR China; 3 Department of Laboratorial Science and Technology, School of Public Health, Peking University, Beijing, PR China; 4 Beijing Key Laboratory of Vector Borne and Natural Focus Infectious Diseases, Beijing, PR China; University of Texas Medical Branch, UNITED STATES

## Abstract

Severe fever with thrombocytopenia syndrome (SFTS) is an emerging infectious disease that is caused by a novel bunyavirus SFTSV. Currently our knowledge of the host-related factors that influence the pathogenesis of disease is inadequate to allow prediction of fatal outcome. Here we conducted a prospective study of the largest database on the SFTS patients, to identify the presence of comorbidities in SFTS, and estimate their effect on the fatal outcome. Among 2096 patients eligible for inclusion, we identified nine kinds of comorbidities, from which hyperlipidemia (12.2%; 95% CI: 10.8%–13.6%), hypertension (11.0%; 95% CI: 9.6%–12.3%), chronic viral hepatitis (CVH) (9.3%; 95% CI: 8.1%–10.5%), and diabetes mellitus (DM) (6.8%; 95% CI: 5.7%–7.9%) were prevalent. Higher risk of death was found in patients with DM (adjusted OR = 2.304; 95% CI: 1.520–3.492; P<0.001), CVH (adjusted OR = 1.551; 95% CI: 1.053–2.285; P = 0.026) and chronic obstructive pulmonary diseases (COPD) (adjusted OR = 2.170; 95% CI: 1.215–3.872; P = 0.009) after adjusting for age, sex, delay from disease onset to admission and treatment regimens. When analyzing the comorbidities separately, we found that the high serum glucose could augment diseases severity. Compared to the group with max glucose < 7.0 mmol/L, patients with glucose between 7.0–11.1 mmol/L and glucose ≥11.1 mmol/L conferred higher death risk, with the adjusted OR to be 1.467 (95% CI: 1.081–1.989; P = 0.014) and 3.443 (95% CI: 2.427–4.884; P<0.001). Insulin therapy could effectively reduce the risk of severe outcome in DM patients with the adjusted OR 0.146 (95% CI: 0.058–0.365; P<0.001). For CVH patients, severe damage of liver and prolongation of blood coagulation time, as well as high prevalence of bleeding phenotype were observed. These data supported the provocative hypothesis that treating SFTS related complications can attain potentially beneficial effects on SFTS.

## Author summary

SFTS now brings about a substantial global public health concern. Preexisting chronic conditions were thought to increase risk of severe SFTSV infections, however with sparse data mining efforts. In this study, we quantified the frequency of chronic comorbidities in SFTS, estimated their contribution to disease severity, and separately evaluated the effect from diabetes mellitus and chronic viral hepatitis on resulting in fatal outcome.

## Introduction

SFTS is an emerging infectious disease that was first reported in 2009 in rural areas in central China. SFTSV is principally transmitted to human by tick bites in natural foci, with a possible human-to-human transmission through contacts with infected blood or body fluid [1]. After infection, patients experienced an extensively wide clinical spectrum, with some experiencing self-limiting clinical course, while approximately 16.2% (95% CI: 14.6%-17.8%) developing fatal outcome [2]. Until now, the risk factors for the fatal outcome and pathogenesis mechanisms underlying the fast proceeding to fatal SFTS remained sparsely investigated [2–4]. As has been displayed widely, numerous factors can influence the outcome of viral infections, including but not restricted to pathogen factors, host genetic susceptibility, host immunity response, host comorbidity conditions, and the effects of therapy [5–7]. All of these factors play in a complex way to determine the final outcome after viral infection. For SFTSV infection, our knowledge of the host-related factors that influence the pathogenesis of disease is far from adequate to allow prediction of fatal outcome. Only older age has been associated with higher risk of fatal outcome with consistent conclusion [8–10]. Preexisting chronic conditions, on the other hand, although long been considered to increase risk of death in a range of viral diseases, were rarely studied in SFTS [11, 12]. Moreover, the specific classification of the diseases was not assigned, therefore making the identification of high-risk population unlikely to attain.

In the current study, we are designed to characterize the prevalence of the common preexisting comorbidities related to the metabolic syndromes-associated diseases, including hyperlipidemia, hypertension, chronic viral hepatitis (CVH), diabetes mellitus (DM), cerebral ischemic stroke, heart diseases, chronic obstructive pulmonary diseases (COPD), pulmonary tuberculosis and cancer in SFTSV infections and to evaluate their effect on diseases outcome. Considering the potential interaction between SFTS and comorbidities such as DM, hyperlipidemia, hypertension on resulting in endothelial dysfunction [13–17], we further measured the level of adhesion factors in patients with or without comorbidities as an indicator of endothelial activation/dysfunction.

## Methods

### Study hospital and patients definition

The study was performed on a prospectively observed cohort of SFTSV infected patients who were recruited in People's Liberation Army (PLA) 154 hospital (now named as The 990 Hospital of Chinese People's Liberation Army Joint Logistic Support Force). The basic information on the cohort has been described in our previous study [2]. Briefly, the hospital is located in Xinyang city, which is located at the southern part of Henan Province, bordering the provinces of Anhui and Hubei to the east and south respectively, representing one of the most severely inflicted cities in central China. Since the beginning of SFTS surveillance in 2011 till 2017, the hospital had diagnosed and treated the largest number (over 30%) of total SFTS cases in China [18]. All the 2096 laboratory-confirmed SFTSV patients were used for the analysis, who met one or more of the following criteria: (1) a positive SFTSV culture (2) a positive result for SFTSV RNA by real-time RT-PCR assay (3) seroconversion or ≥4 fold increase of antibody titers between 2 serum samples collected at least 2 weeks apart.

### Data collection

The medical record of all the hospitalized patients was maintained in an electronic system with logic error correction function, ensuring the credibility of the data. For the current research, a medical record review was performed to collect the information on demographic characteristics, preexisting comorbidities, clinical information, laboratory test results and treatment regimens during the entire hospitalization. The clinical information mainly included symptoms and signs that were recorded from the daily physical examination. The extracted laboratory results mainly included hematology, clinical chemistry, urinalysis and live function examination, which were prescribed on hospital admission and during the hospitalization. Other laboratory indicator included blood cultures, HIV, HBV, HCV-specific antigen and antibody testing, electrocardiogram as well as chest radiograph test, which were prescribed on hospital admission. These data were drawn from the database by a group of trained physicians using a standardized format and entered into an EpiData database. The data were further reviewed for accuracy and consistency by a second group of epidemiologists. For the patients who had missing information, a trained study staff interviewed the patients or their family using a standardized supplemental questionnaire.

### Definition of comorbidities

The comorbidities that were used for the current analysis included diabetes mellitus (both type I and type II, ICD-10 E14.8), hypertension (ICD-10 I10.X02), hyperlipidemia (ICD-10 E78.500), chronic virus hepatitis (both HBV and HCV, ICD-10 B18.951), cerebral ischemic stroke (ICD-10 I64.X04), chronic obstructive pulmonary diseases (ICD-10 J44.900), pulmonary tuberculosis (ICD-10 B90.901), cancer (ICD-10 C00-C97), heart diseases (ICD-10 I51.900, due to the small sample size of individual conditions, cardiac heart failure, coronary atherosclerotic heart disease, arrhythmia and other heart diseases were combined into this category).

### Measurement of viral load, adhesion factors and cytokines

The virus load was determined using real-time reverse transcriptase polymerase-chain-reaction (RT-PCR) targeting the same gene segment. Serum levels of ten adhesion factors were determined by the ProcartaPlex multiplex immunoassays panels (Affymetrix, USA) according to the manufacturer instructions. The measurement of 25 cytokines levels was performed for the serum samples of the survived patient by using Cytokine Human 25-Plex Panel (Life Technologies, USA). The serum samples tested adhesion factors and cytokines were collected on admission and all were within seven days after the onset of disease.

### Statistical analysis

Continuous variables were summarized as means and standard deviations (SD) or as medians and interquartile range (IQR). Categorical variables were summarized as frequencies and proportions. An independent t test, a χ^2^ test, a Fisher exact test, or a nonparametric test was used where appropriate to calculate the differences between groups.

Logistic regression model was applied to explore the association between comorbidities and clinical information or fatal outcome. The generalized estimating equation (GEE) was constructed to compare the laboratory parameters that were evaluated over time. Cytokine and adhesion factors were compared between groups after performing 10 logarithmic transformations using generalized linear model (GLM). Age, sex, time from disease onset to admission and treatment regimens (ribavirin, corticosteroid and immunoglobulin) were adjusted in the above models. Odds ratios (ORs) and their 95% confidence intervals (CIs) were estimated. A two-sided P < 0.05 was considered statistically significant. All analyses were performed using Stata 14.0 (Stata Corp LP, College Station, TX, USA).

### Ethical review

The study protocol was approved by the human ethics committee of the hospital PLA 154. Written or verbal informed consent had been obtained from all the patients or from parents/guardians on behalf of all pediatric participants.

## Results

### Basic information of the case cohort

**Fig 1:**
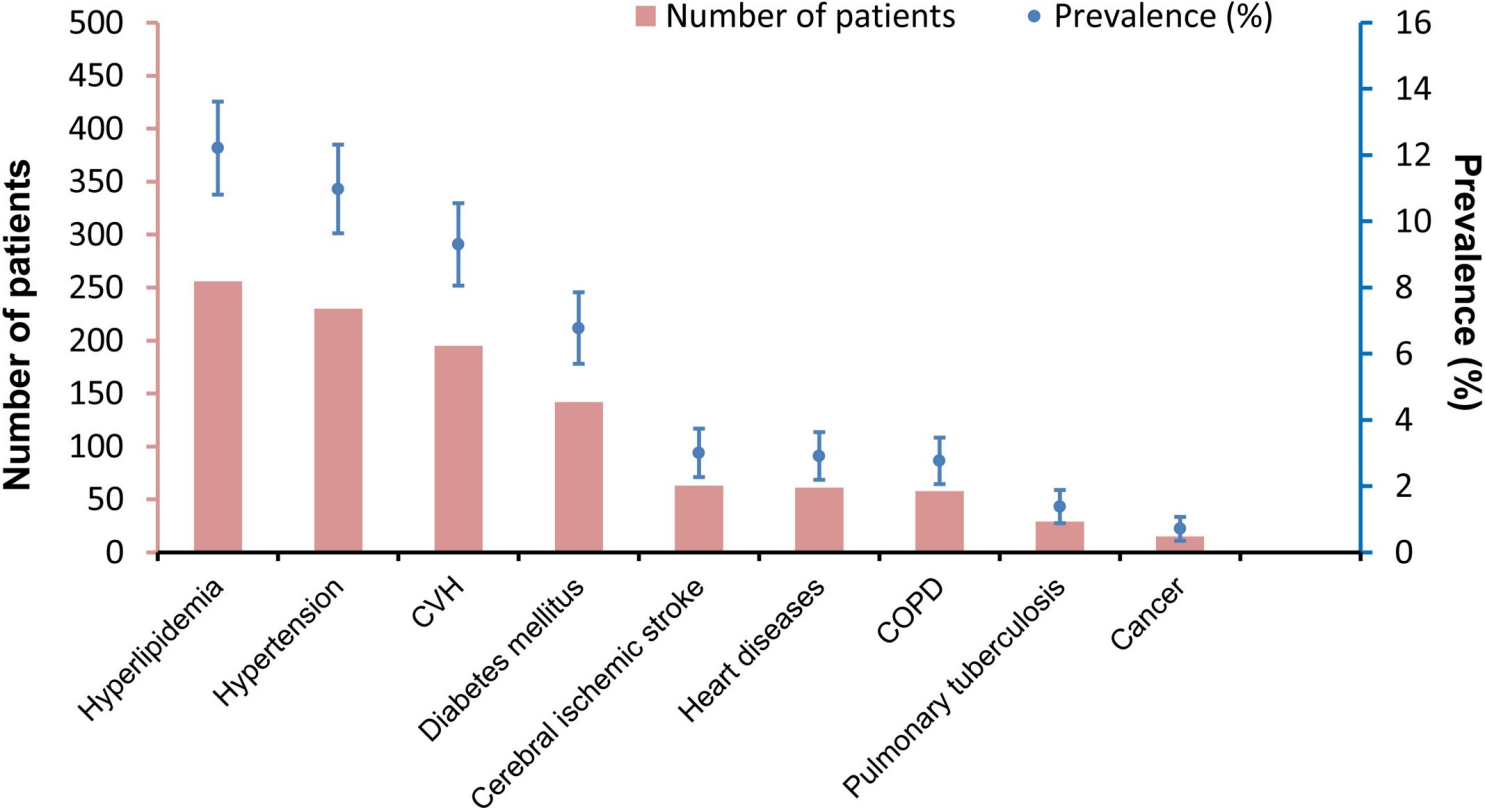
The prevalence of comorbidities in SFTS patients. CVH: chronic viral hepatitis. COPD: chronic obstructive pulmonary diseases.

A total of 2096 laboratory-confirmed SFTS patients who were hospitalized from 2011 to 2017 were used for analysis [2]. The mean (SD) of the age was 61.4 (12.2) years old, and 1239 (59.1%) were female. Overall, the presence of preexisting comorbidities was reported in 779 (37.2%) of the patients. Hyperlipidemia was the most prevalent comorbidity (n = 256; 12.2%; 95% CI: 10.8%-13.6%), followed by hypertension (n = 230; 11.0%; 95% CI: 9.6%-12.3%), CVH (n = 195; 9.3%; 95% CI: 8.1%-10.5%), and DM (n = 142; 6.8%; 95% CI: 5.7%-7.9%) and other diseases (Fig 1). The SFTS patients with the comorbidities had older age and longer time from disease onset to admission compared those without (both P<0.001) (Table 1). For the 195 patients with CVH, 179 (91.8%) were infected with HBV, 15 (7.7%) with HCV and 1 (0.5%) with both infection. Among the patients, 179 had two kinds of comorbidities and 41 had three or more, mostly observed between hyperlipidemia and others (Table 2).

**Table 1:** The demographic characteristics and clinical manifestations of SFTS patients with or without comorbidities.

Characteristics	Comorbidities			
	With	Without	P value	Adjusted
	(n = 779)	(n = 1317)		P value^#^
**Demographic characteristics**				
Male gender/ No. (%)	317 (40.7)	540 (41.0)	0.889 ^a^	
Age, years, mean±SD	64.4±10.1	59.6±13.1	<0.001^b^	
Time from disease onset to admission, days, median (IQR)	6 (4–7)	5 (4–7)	<0.001^c^	
**Clinical manifestations**				
Fever	779 (100)	1317 (100)	NA	NA
Dizziness	184 (23.6)	245 (18.6)	0.006 ^a^*	0.001*
Headache	124 (15.9)	154 (11.7)	0.006 ^a^*	0.003*
Chills	107 (13.7)	146 (11.1)	0.072 ^a^	0.048*
Myalgias	644 (82.7)	1080 (82.0)	0.700 ^a^	0.918
Lymphadenopathy	400 (51.4)	731 (55.5)	0.065 ^a^	0.105
Gastrointestinal symptoms	744 (95.5)	1230 (93.4)	0.046 ^a^*	0.034*
Diarrhoea	217 (27.9)	383 (29.1)	0.549 ^a^	0.447
Abdominal pain	63 (8.1)	80 (6.1)	0.077 ^a^	0.075
Vomiting	296 (38.0)	464 (35.2)	0.203 ^a^	0.130
Nausea	561 (72.0)	943 (71.6)	0.839 ^a^	0.742
Anorexia	648 (83.2)	978 (74.3)	<0.001^a^	<0.001
Respiratory symptoms	469 (60.2)	655 (49.7)	<0.001^a^	0.002*
Dyspnoea	91 (11.7)	96 (7.3)	0.001 ^a^*	0.032*
Sputum	357 (45.8)	477 (36.2)	<0.001^a^	0.004*
Cough	446 (57.3)	627 (47.6)	<0.001^a^	0.004*
Neurological symptoms	276 (35.4)	276 (21.0)	<0.001^a^	<0.001
Coma	90 (11.6)	75 (5.7)	<0.001^a^	0.001*
Lethargy	56 (7.2)	52 (4.0)	0.001^a^*	0.069
Confusion	179 (23.0)	180 (13.7)	<0.001^a^	0.003*
Dysphoria	152 (19.5)	139 (10.6)	<0.001^a^	<0.001
Convulsion	161 (20.7)	150 (11.4)	<0.001^a^	<0.001
Haemorrhagic symptoms	320 (41.1)	415 (31.5)	<0.001^a^	0.003*
Ophthalmorrhagia	4 (0.5)	8 (0.6)	1.000 ^a^	0.709
Ecchymosis	221 (28.4)	259 (19.7)	<0.001^a^	0.001*
Haematemesis	17 (2.2)	30 (2.3)	0.886 ^a^	0.675
Epistaxis	7 (0.9)	9 (0.7)	0.584 ^a^	0.882
Melena	46 (5.9)	92 (7.0)	0.335 ^a^	0.081
Haemoptysis	33 (4.2)	44 (3.3)	0.292 ^a^	0.576
Gingival bleeding	107 (13.7)	101 (7.7)	<0.001^a^	<0.001
Petechia	25 (3.2)	22 (1.7)	0.021 ^a^*	0.027*
Macroscopic haematuria	6 (0.8)	1 (0.1)	0.012 ^a^*	0.039*

Note: Data are No.(%) of patients, mean±standard deviation, or median (IQR).

^a^ By means of the χ^2^ test or Fisher exact test.

^b^ By means of the t test.

^c^ By means of the nonparametric test.

* P < 0.05

^#^ Adjusting for age, sex, time from disease onset to admission and treatment regimens (ribavirin, corticosteroid and immunoglobulin) by applying logistic regression model.

**Table 2:** The risk effect of comorbidities on fatal outcome in SFTS patients.

	Outcome					
	Fatal	Survival	OR(95%CI)	P value	Adjusted	Adjusted
					OR(95%CI)^#^	P value^#^
**Without comorbidities**	165 (12.5)	1152 (87.5)	Reference		Reference	
**One kind of comorbidity**	123 (22.0)	436 (78.0)	1.970 (1.521–2.550)	<0.001	1.631 (1.233–2.159)	<0.001
Hyperlipidemia	19 (13.2)	125 (86.8)	1.061 (0.638–1.766)	0.819	0.912 (0.520–1.601)	0.749
Hypertension	24 (21.2)	89 (78.8)	1.883 (1.166–3.041)	0.010*	1.610 (0.956–2.710)	0.073
Chronic viral hepatitis	26 (21.3)	96 (78.7)	1.891 (1.190–3.004)	0.007*	2.006 (1.215–3.311)	0.007*
Diabetes	18 (27.7)	47 (72.3)	2.674 (1.516–4.715)	0.001*	2.462 (1.310–4.630)	0.005*
COPD	16 (44.4)	20 (55.6)	5.585 (2.837–10.996)	<0.001	2.715 (1.295–5.691)	0.008*
**Two kinds of comorbidities**	40 (22.4)	139 (77.6)	2.009 (1.363–2.961)	<0.001	1.447 (0.955–2.194)	0.082
Hypertension & Hyperlipidemia	7 (26.9)	19 (73.1)	2.572 (1.065–6.213)	0.036*	2.101 (0.822–5.370)	0.121
Diabetes & Hyperlipidemia	7 (38.9)	11 (61.1)	4.443 (1.699–11.622)	0.002*	3.545 (1.184–10.620)	0.024*
Chronic viral hepatitis & Hyperlipidemia	1 (5.3)	18 (94.7)	0.388 (0.051–2.925)	0.358	0.309 (0.040–2.409)	0.262
Chronic viral hepatitis & Hypertension	3 (20.0)	12 (80.0)	1.745 (0.487–6.250)	0.392	1.010 (0.257–3.975)	0.989
Diabetes & Hypertension	2 (12.5)	14 (87.5)	0.997 (0.225–4.428)	0.997	0.921 (0.198–4.287)	0.917
Diabetes & Chronic viral hepatitis	5 (45.5)	6 (54.5)	5.818 (1.756–19.278)	0.004*	4.792 (1.345–17.077)	0.016*
**Three or more kinds of comorbidities**	12 (29.3)	29 (70.7)	2.889 (1.446–5.773)	0.003*	2.399 (1.128–5.106)	0.023*

^#^Adjusting for age, sex, time from disease onset to admission and treatment regimens (ribavirin, corticosteroid and immunoglobulin) by applying logistic regression model. The COPD was not listed in the two kinds of comorbidities due to the small sample size.

* P < 0.05.

### Association between comorbidities and risk of fatal outcome

**Fig 2:**
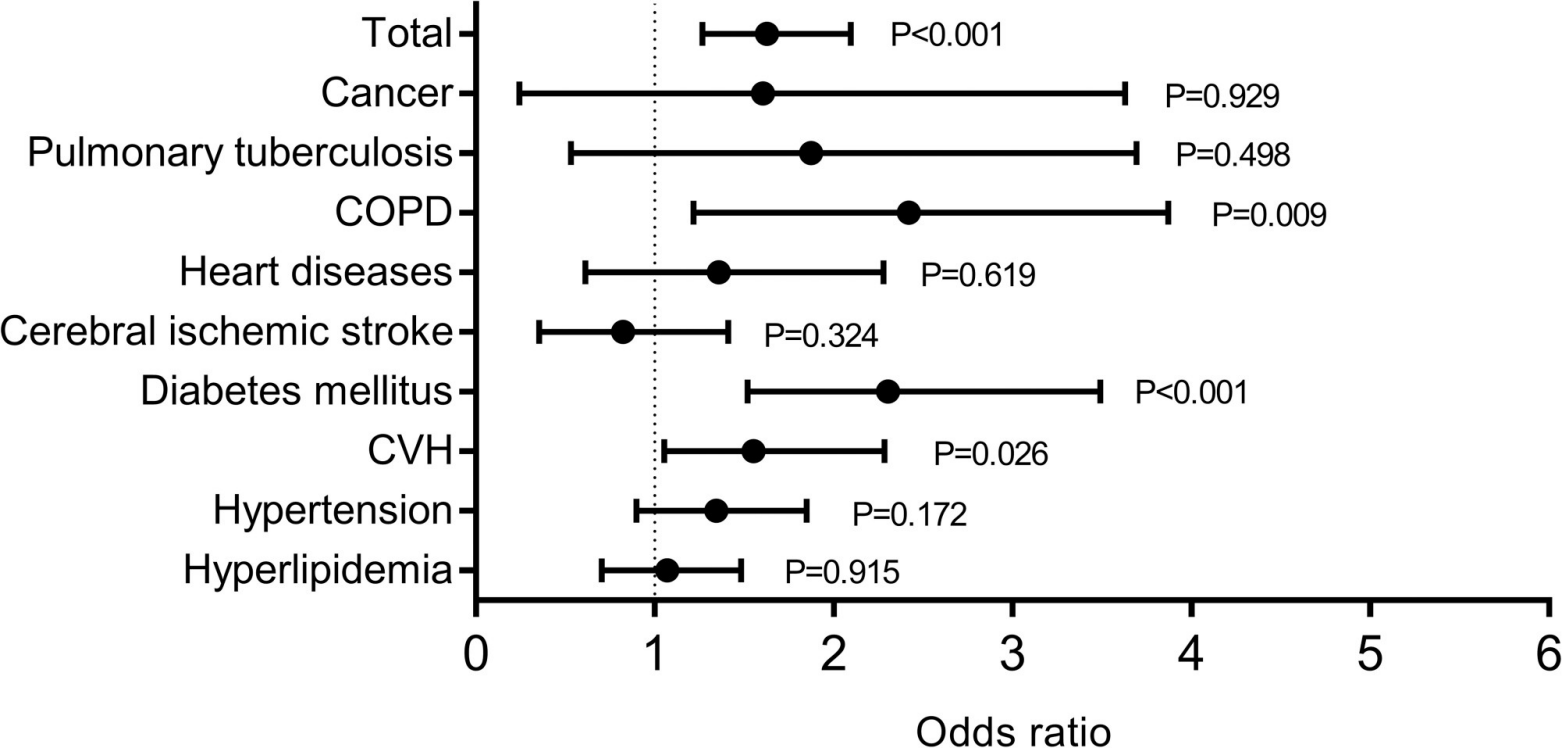
The association between comorbidities and fatal outcome. Each of the comorbidities and as a whole were analyzed for their association with death after adjusting for age, sex, time from disease onset to admission and treatment regimens (ribavirin, corticosteroid and immunoglobulin) by applying logistic regression model.

The case fatality rate (CFR) among the patients with any kind of comorbidity was 22.5% (175/779), significantly higher than those without (12.5%; 165/1317; adjusted OR = 1.628; 95% CI: 1.265–2.096; P<0.001) (S1 Table). When the comorbidities were assessed individually, hypertension, DM, CVH and COPD were significantly associated with the development of fatal outcome (all P<0.05). However, after adjusting the effect from age, sex, delay from disease onset to admission and treatment regimens (ribavirin, corticosteroid and immunoglobulin), the significance only remained for DM (adjusted OR = 2.304; 95% CI: 1.520–3.492; P<0.001), CVH (adjusted OR = 1.551; 95% CI: 1.053–2.285; P = 0.026) and COPD (adjusted OR = 2.170; 95% CI: 1.215–3.872; P = 0.009) (Fig 2 and S1 Table). The presence of over one kind of comorbidity was associated with enhanced risk of death, with the coexistence of DM & CVH having significantly higher odds ratio of developing fatal outcome (adjusted OR = 4.792; 95% CI: 1.345–17.077; P = 0.016), in comparison with those without any comorbidity, thus indicating an interaction between them (Table 2).

We applied a logistic regression model with stepwise method to adjust the potential interaction effects that might be derived from inter-comorbidities. The analysis showed three significant comorbidities in the model that attained higher risk of death: the presence of DM (adjusted OR = 2.328; 95% CI: 1.534–3.532; P<0.001), CVH (adjusted OR = 1.557; 95% CI: 1.056–2.296; P = 0.025), and COPD (adjusted OR = 2.138; 95% CI: 1.195–3.825; P = 0.010).

### Clinical manifestations and laboratory assessments in the patients with comorbidities as a whole

**Fig 3:**
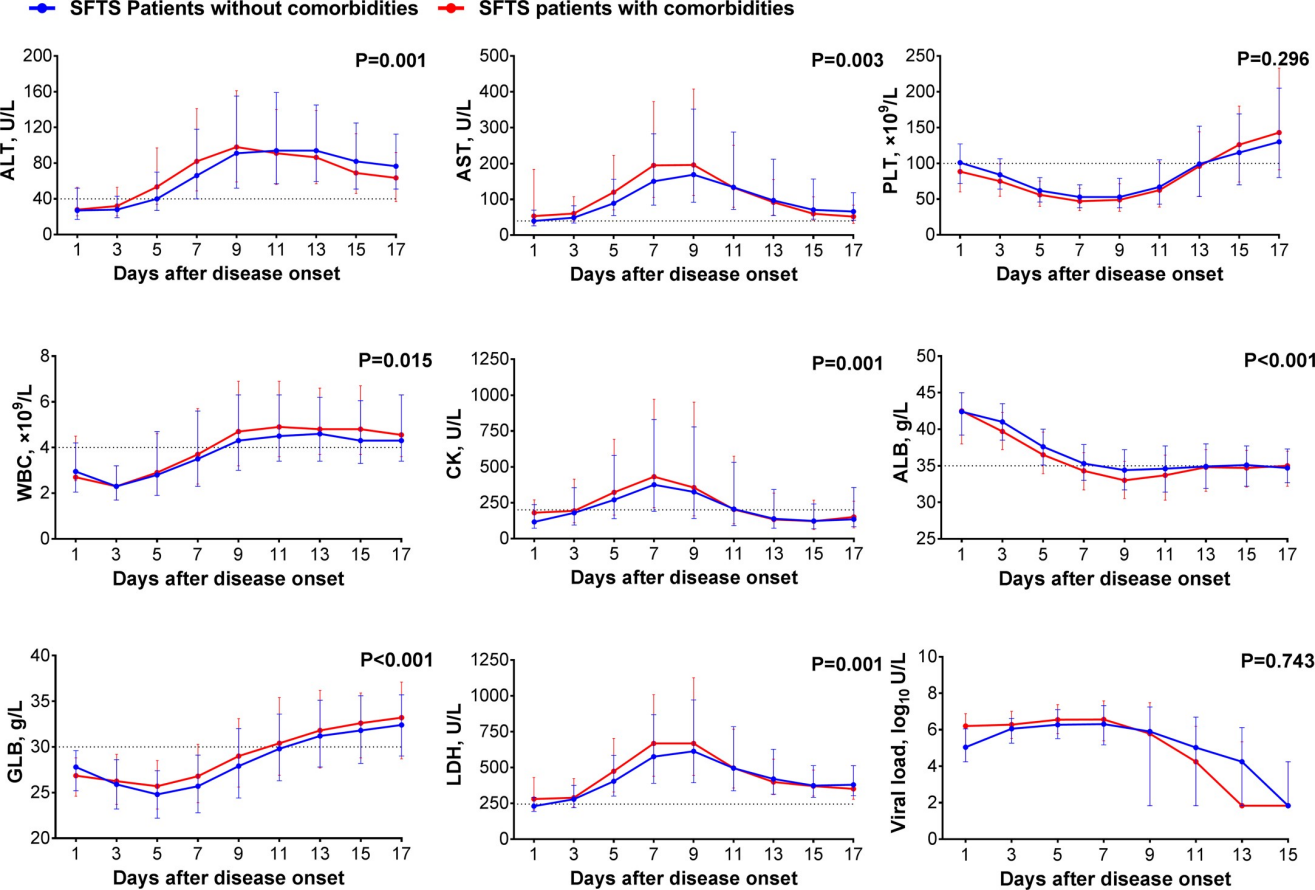
Comparison of laboratory parameters between SFTS patients with or without comorbidities. The effect was attained after adjusting for age, sex, time from disease onset to admission and treatment regimens (ribavirin, corticosteroid and immunoglobulin) by applying GEE. ALT, alanine transaminase; AST, aspartate aminotransferase; PLT, platelet; WBC, white blood cell; CK, creatine kinase; ALB, albumin; GLB, globulin; LDH, lactate dehydrogenase; Viral load, SFTSV viral load. For undetectable viral load, a value of half of the limit of detection was assigned.

Clinical manifestations and laboratory assessments were compared between two groups. At presentation, three of the commonly seen signs or symptoms, including dizziness, headache, chills and gastrointestinal symptoms were more frequently observed in SFTS patients with comorbidities than those without. Severe complications, including respiratory symptoms, neurological symptoms and haemorrhagic symptoms developed with higher frequency in SFTS patients with any kind of comorbidities than those without (Table 1). The GEE analysis displayed levels of alanine transaminase (ALT), aspartate aminotransferase (AST), white blood cell (WBC), creatine kinase (CK), globulin (GLB) and lactate dehydrogenase (LDH) were significantly elevated, while the level of albumin (ALB) significantly decreased among the SFTS patients with comorbidities. No differences in SFTSV viral load or platelet counts were observed between the two groups (Fig 3).

### Clinical manifestations and laboratory assessments in the patients with DM

**Fig 4:**
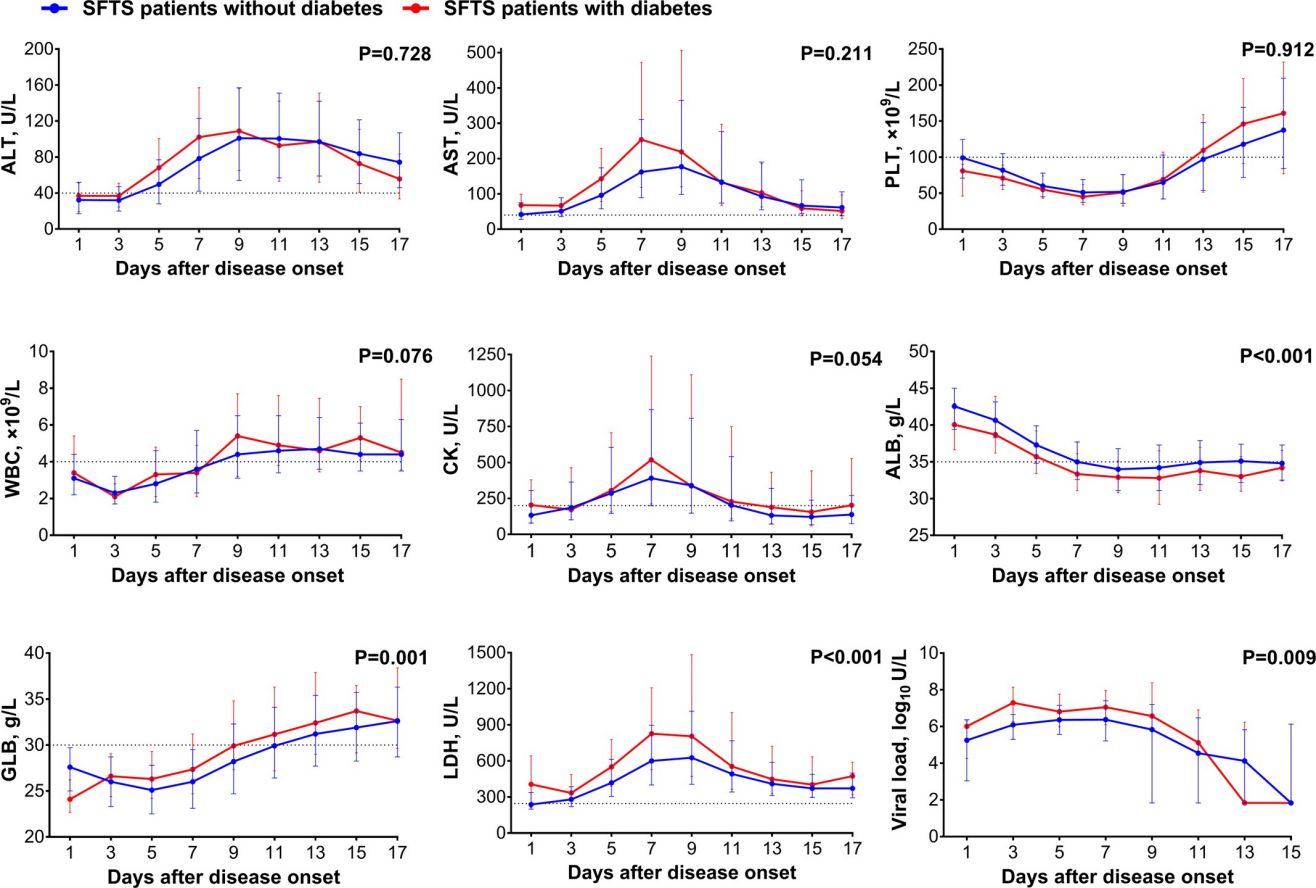
Comparison of laboratory parameters between SFTS patients with or without diabetes. The effect was attained after adjusting for age, sex, time from disease onset to admission and treatment regimens (ribavirin, corticosteroid and immunoglobulin) by applying GEE. ALT, alanine transaminase; AST, aspartate aminotransferase; PLT, platelet; WBC, white blood cell; CK, creatine kinase; ALB, albumin; GLB, globulin; LDH, lactate dehydrogenase; Viral load, SFTSV viral load. For undetectable viral load, a value of half of the limit of detection was assigned.

Altogether 142 patients with DM and 1954 without were compared for their clinical manifestations and laboratory indicators. Most of the initial symptoms and signs were reported from two groups with similar frequencies (S2 Table). On the other hand, respiratory and neurological complications were more likely to develop in the patients with DM than in patients without. The dynamic profiles of laboratory parameters during the whole course were similar between two groups, except three higher levels of laboratory indicators (GLB, LDH and SFTSV viral loads) and lower levels of ALB in the patients with DM than those without (Fig 4).

**Fig 5:**
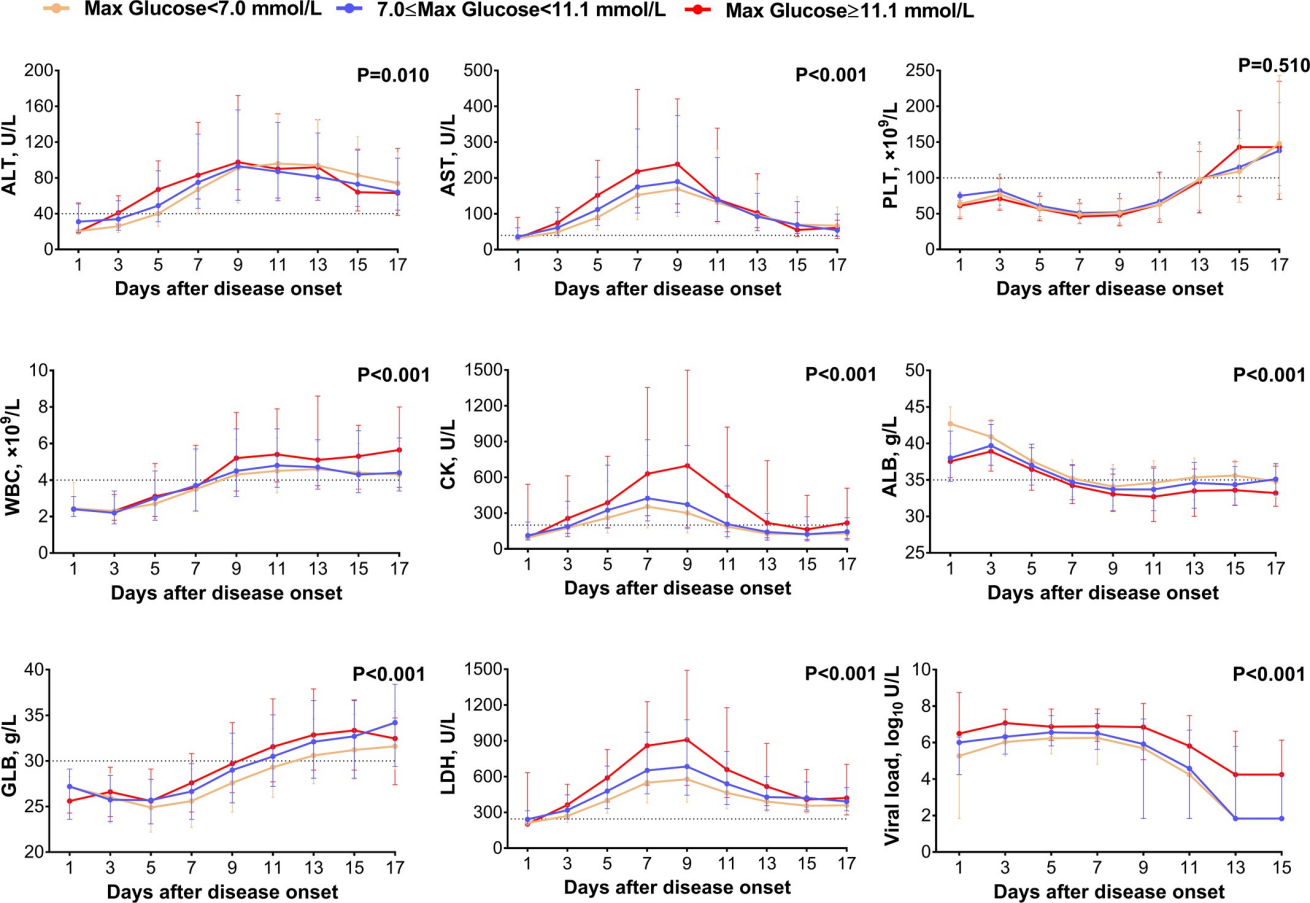
Comparison of laboratory parameters between the SFTS patients with different levels of max glucose. The effect was attained after adjusting for age, sex, time from disease onset to admission and treatment regimens (ribavirin, corticosteroid and immunoglobulin) by applying GEE. ALT, alanine transaminase; AST, aspartate aminotransferase; PLT, platelet; WBC, white blood cell; CK, creatine kinase; ALB, albumin; GLB, globulin; LDH, lactate dehydrogenase; Viral load, SFTSV viral load. For undetectable viral load, a value of half of the limit of detection was assigned.

**Fig 6:**
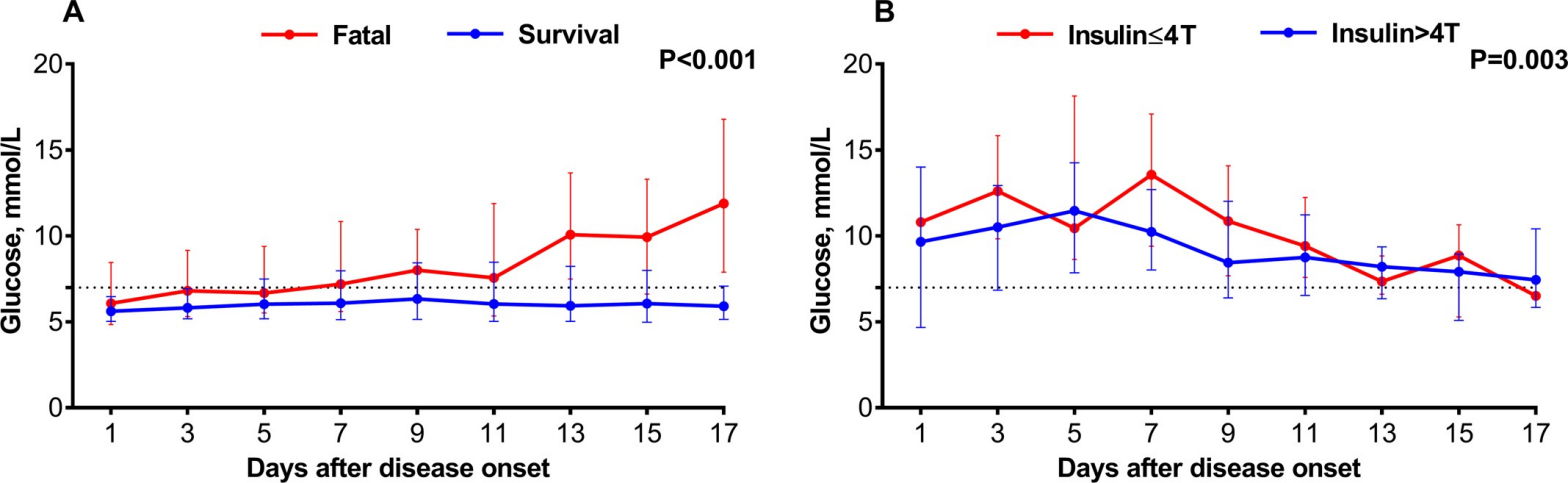
**Comparison of glucose among SFTS patients stratified by outcome(A) and insulin using times (B).** The effect was attained after adjusting for age, sex, time from disease onset to admission and treatment regimens (ribavirin, corticosteroid and immunoglobulin) by applying GEE.

When the patients were further grouped according to the maximum glucose level during the whole hospitalization (S3 Table), a dose dependent effect was displayed as the decrease in ALB, together with elevation in AST, ALT, WBC, CK, GLB, LDH and SFTSV viral loads were negatively correlated with the glucose level (Fig 5). Moreover, the glucose level significantly affected the risk of death (Fig 6A). Compared to the group with glucose < 7.0 mmol/L, patients with glucose between 7.0–11.1 mmol/L and glucose ≥11.1 mmol/L had higher death risk, with the adjusted OR estimated to be 1.467 (95% CI: 1.081–1.989; P = 0.014) and 3.443 (95% CI: 2.427–4.884; P<0.001), respectively (Table 3). The DM patients who received insulin therapy over four times had significant lower glucose level (S4 Table and Fig 6B), which conferred a significantly reduced risk of fatal outcome (adjusted OR = 0.146; 95% CI: 0.058–0.365; P<0.001) (Table 3).

**Table 3:** The association between fatal outcome and glucose level or insulin using times.

Variable	Outcome			
	Fatal	Survival	Adjusted OR	Adjusted
			(95%CI)	P value^#^
**Glucose, mmol/L**	n = 318	n = 1622		
<7.0	134 (42.1)	1042 (64.2)	Reference	
7.0–11.1	97 (30.5)	424 (26.1)	1.467 (1.081–1.989)	0.014*
≥11.1	87 (27.4)	156 (9.6)	3.443 (2.427–4.884)	<0.001
**Insulin use times**	n = 43	n = 99		
≤4T	32 (74.4)	32 (32.3)	Reference	
>4T	11 (25.6)	67 (67.7)	0.146 (0.058–0.365)	<0.001

^#^Adjusting for age, sex, time from disease onset to admission and treatment regimens (ribavirin, corticosteroid and immunoglobulin) by applying logistic regression model.

* P<0.05

**Fig 7:**
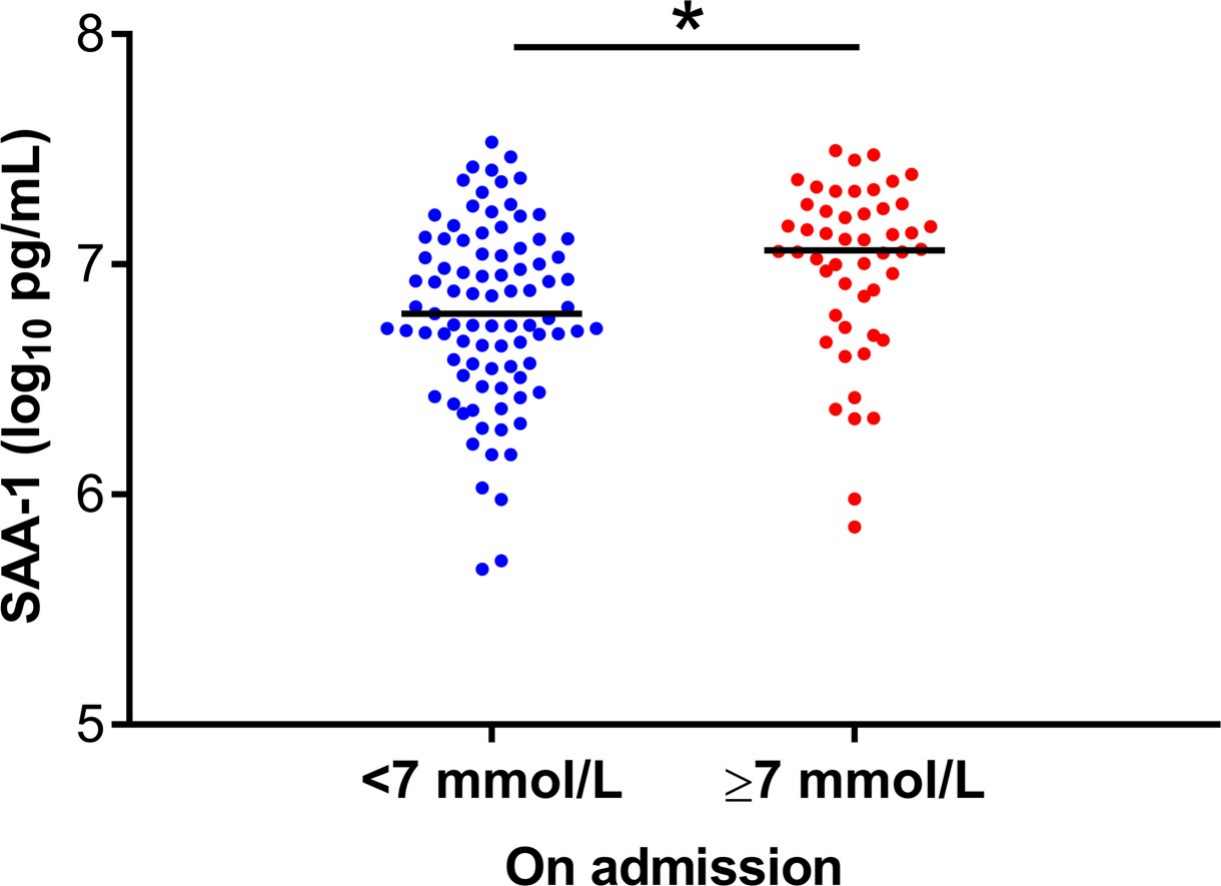
Comparison of adhesion factors among SFTS patients with different glucose levels on admission. The effect was attained after adjusting for age, sex and time from disease onset to admission by applying GLM. SAA-1, serum amyloid antigen 1. The X axis was the glucose level on admission. * P < 0.05, ** P < 0.01, *** P < 0.001.

**Fig 8:**
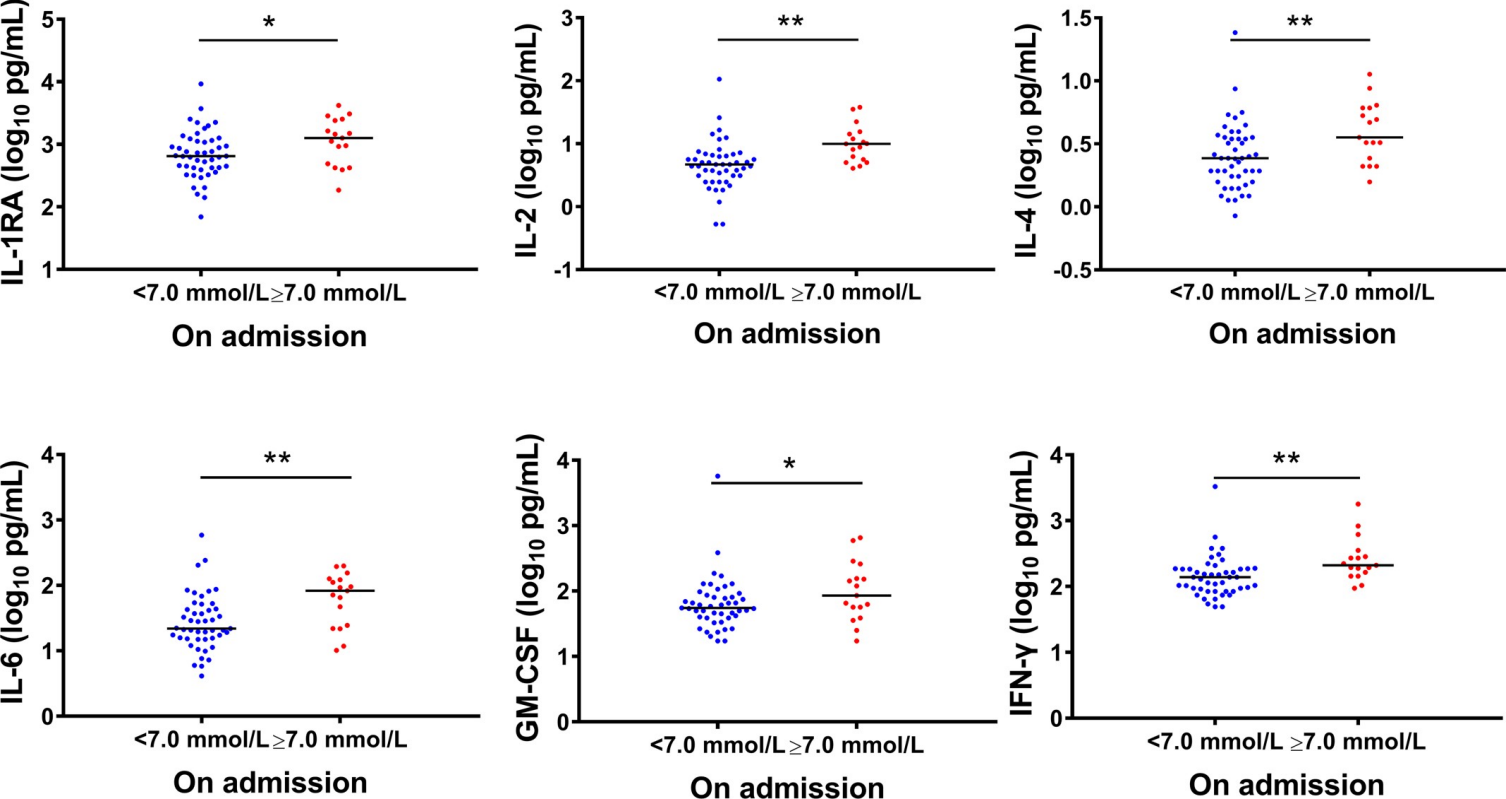
Comparison of cytokines among SFTS patients with different glucose levels on admission. The effect was attained after adjusting for age, sex and time from disease onset to admission by applying GLM. GM-CSF, granulocyte macrophage-stimulating factor; IFN-γ, Interferon-γ. The X axis was the glucose level on admission. * P < 0.05, ** P < 0.01, *** P < 0.001.

Totally 139 patients had ten adhesion factors evaluated, including 50 patients with glucose level ≥ 7.0 mmol/L on admission (S5 Table). Only serum amyloid antigen 1(SAA-1) was found to be elevated in the patients with glucose ≥ 7.0 mmol/L than those with glucose <7.0 mmol/L (Fig 7). Altogether 64 SFTS patients had their serum cytokines measured on admission, including 17 patients with glucose exceeding 7.0 mmol/L (S6 Table). Six of the 25 tested cytokine, including Interleukin-1RA (IL-1RA), Interleukin-2 (IL-2), IL-4, IL-6, Granulocyte macrophage-stimulating factor (GM-CSF), Interferon-γ (IFN-γ) were significantly higher in SFTS patients with high glucose level (Fig 8).

### Clinical manifestations and laboratory assessments in patients with CVH

**Fig 9:**
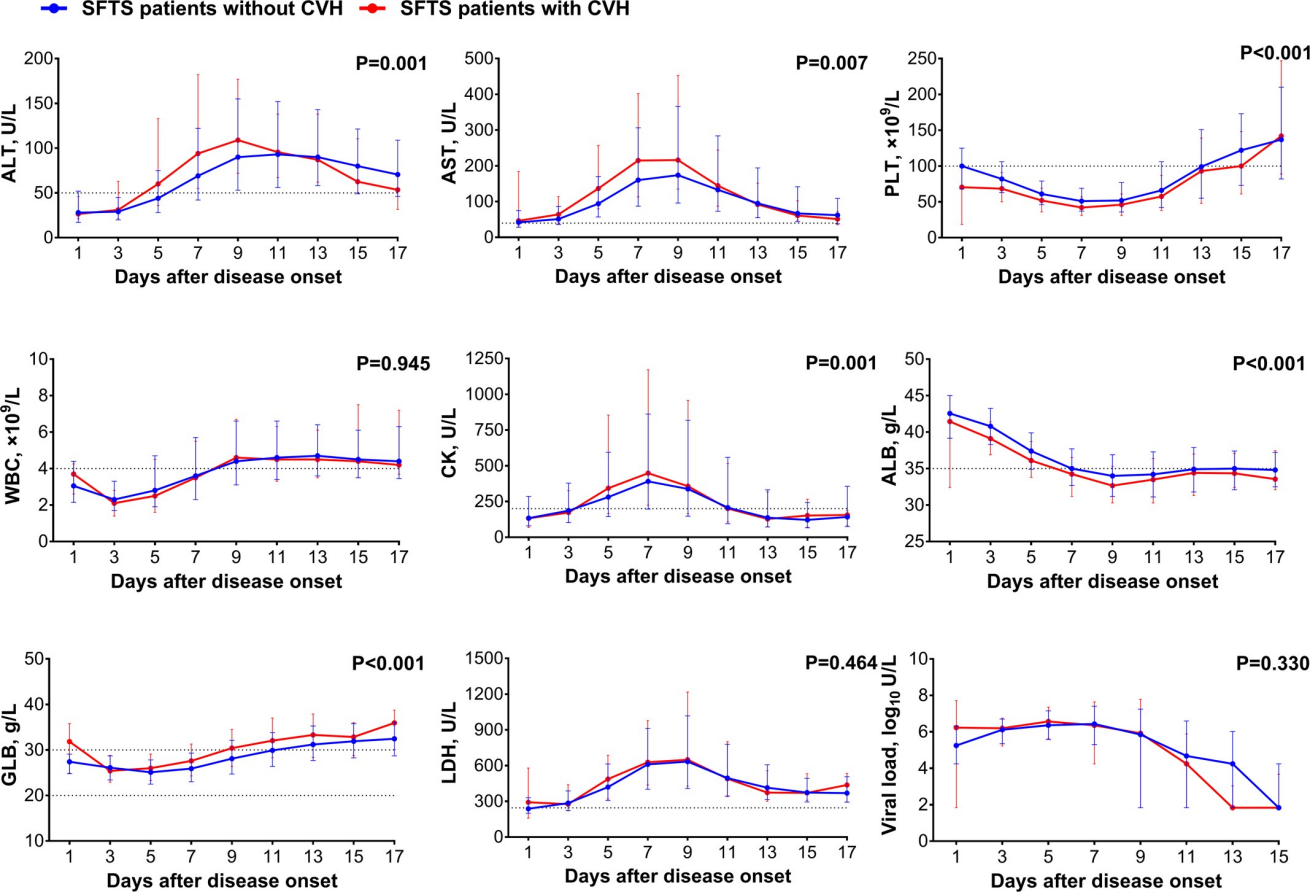
Comparison of laboratory parameters between the SFTS patients with or without CVH. The effect was attained after adjusting for age, sex, time from disease onset to admission and treatment regimens (ribavirin, corticosteroid and immunoglobulin) by applying GEE. ALT, alanine transaminase; AST, aspartate aminotransferase; PLT, platelet; WBC, white blood cell; CK, creatine kinase; ALB, albumin; GLB, globulin; LDH, lactate dehydrogenase; Viral load, SFTSV viral load. For undetectable viral load, a value of half of the limit of detection was assigned.

**Fig 10:**
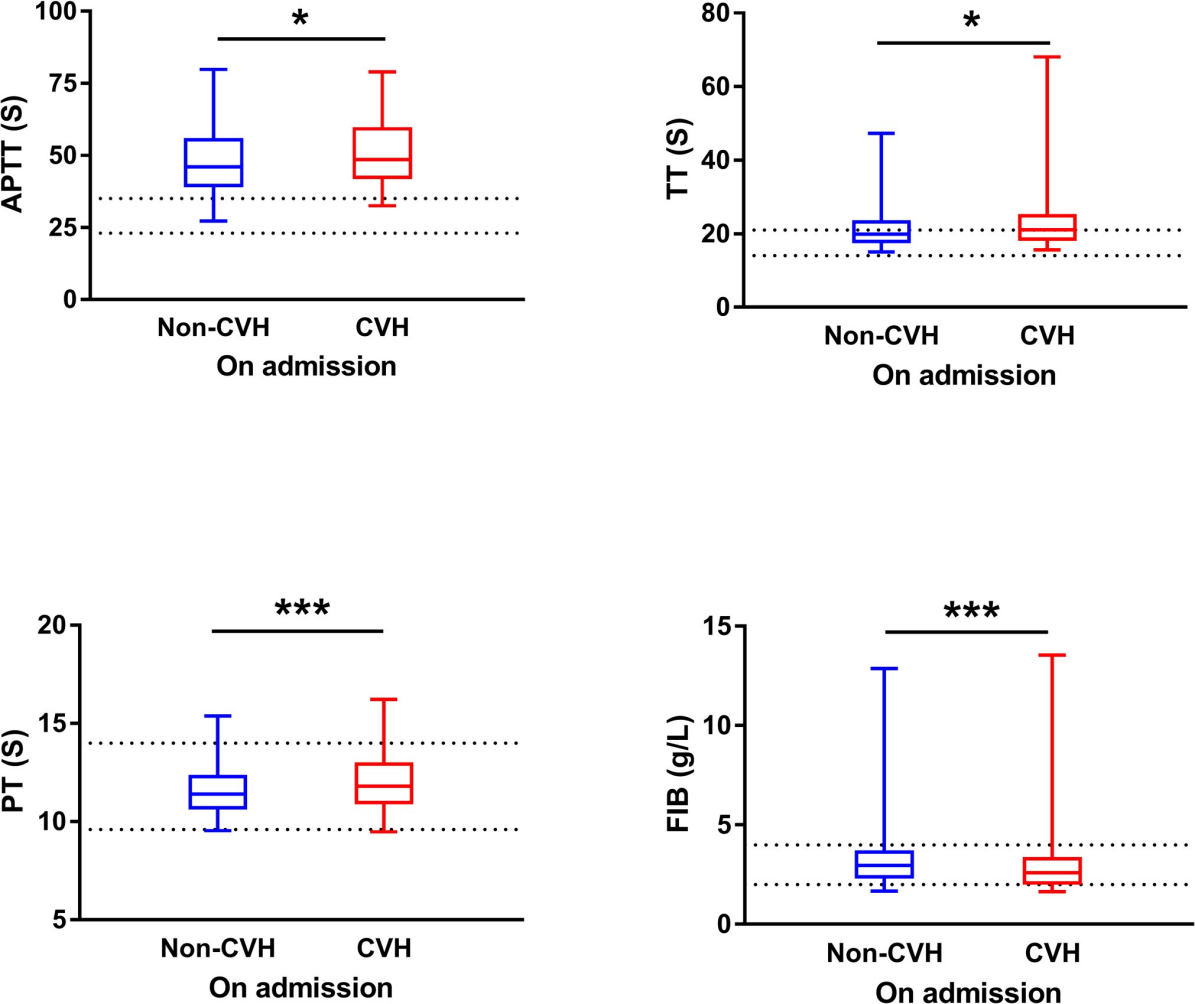
Comparison of blood coagulation function tested in SFTS patients with or without CVH on admission. The effect was attained by applying the nonparametric test. APTT, activated partial thromboplastin time; TT, thrombin time; PT, prothrombin time; FIB, plasma fibrinogen. * P < 0.05, ** P < 0.01, *** P < 0.001.

Altogether 195 patients with CVH and 1901 without were compared for their clinical manifestations and laboratory indicators. Most of the non-specific signs or symptoms were comparable between the CVH and non-CVH groups (S7 Table). Higher frequency of neurological symptoms (33.3% *vs*. 25.6%; adjusted P = 0.045) and haemorrhagic symptoms (45.1% *vs*. 34.0%; adjusted P = 0.017) were disclosed in the CVH group. Higher ALT, AST, CK, GLB concentration, lower platelet counts and ALB level were also related to the presence of CVH in SFTS patients (Fig 9). Totally 98 CVH and 867 non-CVH patients with SFTSV infection had blood coagulation function test on admission available for analysis (S8 Table). The patients with CVH developed more prolongation of the prothrombin time (PT) and activated partial thromboplastin time (APTT) and thrombin time (TT), all indicating occurrence of disseminated intravascular coagulation (DIC). The pattern of these parameters corresponded with decreased platelet and high prevalence of bleeding phenotype (Fig 10).

## Discussion

Over the past few years, several lines of evidence have supported the notion that cardiovascular disease, stroke, diabetes, respiratory diseases and renal disorders may contribute, together with old age, to severe dengue disease [19–21]. Studies on West Nile virus [22], Japanese encephalitis virus [23] infections, and responses to Yellow fever virus vaccination [24], have also supported the pathogenic role of chronic comorbidities in the prognosis of infections. Since the discovery of SFTS, although clinical phenotypes have been developed to differentiate the patients with high risk of death, host factors remained sparsely investigated.

In this study, we demonstrated the frequency of underlying conditions in SFTSV infected patients and determined their role in developing fatal outcome. Hyperlipidemia and hypertension are the most prevalent comorbidity, while DM, CVH and COPD were more prominent in their association with fatal outcome, with 1.551–2.304 fold increase in their risk of death than the SFTS patients without comorbidities. From the perspective of clinical features, neurological manifestation and hemorrhagic signs were more frequently seen in patients with underlying diseases, both contributing to the final fatal outcome.

A common pathogenic feature of SFTS infection is their ability to inhibit the host immune response, characterized by significantly reduced CD3-positive and CD4-positive T lymphocytes than normal [25]. This is consistent with the clinical phenomenon that most infection occurred in the elderly, who are considered to possess compromised T-cell function [26]. The association between DM, CVH and severe diseases might also be related to immune dysfunction. Abnormal innate and adaptive immunity used to be disclosed in DM patients, reflected by alterations in proliferation of T cells and macrophages, and impairment in function of NK cells and B cells in DM patients [27]. CVH, either hepatitis B or hepatitis C, was capable of inhibiting the adaptive or innate immune response [28]. This supported the hypothesis that DM and CVH, in combination with SFTSV infection, might impair the immune system and attenuate anti-inflammatory responses, subsequently resulting in increased level and prolonged duration of viremia, which predispose patients to higher risk of death.

In addition, the preexisting DM is often linked to vascular complications featured by an activation of the inflammation cascade and endothelial dysfunction [13], which are also identified in SFTSV infection [17]. In line with this mechanism, we observed a remarkably enhanced expression of SAA-1, a biomarker of endothelial dysfunction [17], in DM-SFTS than SFTS alone. Cytokine storm had been extensively found to play roles in the pathogenesis of SFTS [29]. Based on the current findings, IL-1RA, IL-2, IL-4, IL-6, GM-CSF and IFN-γ were elevated to remarkably high levels in DM-SFTS patients, likely contributing to the fatal outcome, together with the development of depressed immunity and endothelial dysfunction. All these indicators showed potential to predict adverse outcome. The insulin therapy, on the other hand, had obviously reduced the disease severity. Therefore, it’s justified to actively identify and treat high glucose in SFTSV infection, in order to attain extra benefit of reducing viremia and enhancing disease outcomes.

Differing from DM-SFTS, the CVH-SFTS patients were prone to have higher incidence of bleeding manifestation than those without. In line with these findings, abnormal coagulation factors, including platelet and others, were more frequently seen in CVH-SFTS. An interactive effect on liver damage from CVH and SFTS was observed, as liver function related enzymes, especially AST, ALT and ALB, demonstrated remarkable deviation from normal level, which was indicative of progressive hepatic involvement in those patients. As liver is the primary source for producing coagulation factors [30, 31], it is reasonable to deduce that the interactive effect from SFTSV and hepatitis virus can predispose the patient to more frequent bleeding.

It’s noteworthy that ALB was constantly reduced in patients with DM, or CVH or any kind of comorbidity. ALB is the most abundant protein in plasma, representing the main determinant of plasma oncotic pressure and the main modulator of fluid distribution between body compartments. ALB plays an import role of in plasma leakage that could be a parameter to predict the severity of diseases [32, 33]. Recently, the endothelial dysfunction and plasma leakage had been identified in SFTSV infection and most viral hemorrhagic fever, manifested by fluid loss from the vascular compartment and by decreased level of ALB [20, 34, 35]. It is hypothesized that hypoalbuminemia could be induced from a synergetic effect of comorbidity and SFTSV infection, eventually contributing to the high morbidity and mortality. As such, albumin administration in SFTS patients might be effective in improving the disease outcome.

The study is subject to major limitation that when assessing comorbidities, we did not allow for differentiating between those diagnosed before, after or during the infectious episodes. Therefore, the causality between the conditions and adverse outcome cannot be inferred. However, even in the absence of causal inference between the non-communicable and infectious diseases, these findings may guide clinicians to predict complications, at least partially, based on the presence of comorbidity. In addition, we made no efforts to distinguish type 1 from type 2 diabetes for separate analysis, despite of their differential clinical features and etiological factors. Instead we used the glucose level as major variable to explore the dose effect of glucose on the disease severity. Moreover, the clinical status that were acquired from these patients were only partially used, and due to the high cost of testing adhesion factors and interleukins, we did not evaluate the entire population for these indicators, which might have caused selection bias for the inter-group comparison.

In conclusion, we provided evidence for a higher prevalence of DM, CVH and COPD in fatal SFTS patients, elucidating the possible mechanism that underlies their interactive effect in resulting in adverse outcome. This knowledge might allow clinical physicians to identify the patients with preexisting comorbidities who may progress to a severe course, thereby to adopt aggressive interventions at early infection.

## Supporting information

S1 Table Comparison of clinical outcome between the SFTS patients with or without comorbidities. (DOCX)

S2 Table The characteristics and clinical manifestations of the SFTS patients with or without diabetes. (DOCX)

S3 Table The characteristics of SFTS patients with three levels of maximum glucose during the whole course. (DOCX)

S4 Table The characteristics of DM-SFTS patients stratified by insulin using frequency. (DOCX)

S5 Table The characteristics of SFTS patients who were tested for adhesion factors on admission (stratified by the glucose level on admission). (DOCX)

S6 Table The characteristics of SFTS patients who were tested for cytokines on admission (stratified by the glucose level on admission). (DOCX)

S7 Table The characteristics and clinical manifestations of SFTS patients with or without CVH. (DOCX)

S8 Table The characteristics of SFTS patients who were tested for blood coagulation function on admission (stratified by the presence of CVH). (DOCX)
