# Supplementary material for: Preexisting chronic conditions for fatal outcome among SFTS patients: An observational Cohort Study
Source: PLoS Negl Trop Dis. 2019 May 28;13(5):e0007434. doi: 10.1371/journal.pntd.0007434 (PMC6555536; doi:10.1371/journal.pntd.0007434)
Supplement: S1 Table — (DOCX) [file pntd.0007434.s001.docx]

**S1 Table. Comparison of clinical outcome between the SFTS patients with or without comorbidities.**

| **Comorbidities** | **Fatal** | |  | **Crude** | | |  | **Adjusted^#^** | | |
| --- | --- | --- | --- | --- | --- | --- | --- | --- | --- | --- |
|  | **Yes** | **No** |  | **OR** | **95%CI** | **P value** |  | **OR** | **95%CI** | **P value** |
| **Model 1 for Comorbidities** | | | | | | | | | | |
| Yes | 175 (22.5) | 604 (77.5) |  | 2.023 | 1.600-2.557 | <0.001 |  | 1.628 | 1.265-2.096 | <0.001 |
| No | 165 (12.5) | 1152 (87.5) |  | 1.000 |  |  |  | 1.000 |  |  |
| **Model 2 for Hyperlipidemia** | | | | | | | | | | |
| Yes | 46 (18.0) | 210 (82.0) |  | 1.152 | 0.818-1.622 | 0.419 |  | 1.021 | 0.702-1.484 | 0.915 |
| No | 294 (16.0) | 1546 (84.0) |  | 1.000 |  |  |  | 1.000 |  |  |
| **Model 3 for Hypertension** | | |  |  |  |  |  |  |  |  |
| Yes | 50 (21.7) | 180 (78.3) |  | 1.510 | 1.077-2.115 | 0.017* |  | 1.287 | 0.896-1.849 | 0.172 |
| No | 290 (15.5) | 1576 (84.5) |  | 1.000 |  |  |  | 1.000 |  |  |
| **Model 4 for Chronic viral hepatitis** | | |  |  |  |  |  |  |  |  |
| Yes | 43 (22.0) | 152 (78.0) |  | 1.528 | 1.065-2.191 | 0.021* |  | 1.551 | 1.053-2.285 | 0.026* |
| No | 297 (15.6) | 1604 (84.4) |  | 1.000 |  |  |  | 1.000 |  |  |
| **Model 5 for Diabetes mellitus** | | |  |  |  |  |  |  |  |  |
| Yes | 43 (30.3) | 99 (69.7) |  | 2.423 | 1.659-3.539 | <0.001 |  | 2.304 | 1.520-3.492 | <0.001 |
| No | 297 (15.2) | 1657 (84.8) |  | 1.000 |  |  |  | 1.000 |  |  |
| **Model 6 for Cerebral ischemic stroke** | | |  |  |  |  |  |  |  |  |
| Yes | 11 (17.5) | 52 (82.5) |  | 1.096 | 0.566-2.122 | 0.787 |  | 0.706 | 0.354-1.410 | 0.324 |
| No | 329 (16.2) | 1704 (83.8) |  | 1.000 |  |  |  | 1.000 |  |  |
| **Model 7 for Heart diseases** | | |  |  |  |  |  |  |  |  |
| Yes | 15 (24.6) | 46 (75.4) |  | 1.716 | 0.947-3.110 | 0.075 |  | 1.181 | 0.612-2.279 | 0.619 |
| No | 325 (16.0) | 1710 (84.0) |  | 1.000 |  |  |  | 1.000 |  |  |
| **Model 8 for Chronic obstructive pulmonary diseases** | | | | | |  |  |  |  |  |
| Yes | 25 (43.1) | 33 (56.9) |  | 4.144 | 2.431-7.064 | <0.001 |  | 2.170 | 1.215-3.872 | 0.009* |
| No | 315 (15.5) | 1723 (84.5) |  | 1.000 |  |  |  | 1.000 |  |  |
| **Model 9 for Pulmonary tuberculosis** | | |  |  |  |  |  |  |  |  |
| Yes | 6 (20.7) | 23 (79.3) |  | 1.354 | 0.547-3.350 | 0.513 |  | 1.399 | 0.530-3.694 | 0.498 |
| No | 334 (16.2) | 1733 (83.8) |  | 1.000 |  |  |  | 1.000 |  |  |
| **Model 10 for Cancer** | |  |  |  |  |  |  |  |  |  |
| Yes | 3 (20.0) | 12 (80.0) |  | 1.294 | 0.363-4.609 | 0.691 |  | 0.940 | 0.243-3.631 | 0.929 |
| No | 337 (16.2) | 1744 (83.8) |  | 1.000 |  |  |  | 1.000 |  |  |

^#^Adjusting for age, sex, time from disease onset to admission and treatment regimens (ribavirin, corticosteroid and immunoglobulin) by applying logistic regression model.

*P < 0.05
