# Supplementary material for: Preexisting chronic conditions for fatal outcome among SFTS patients: An observational Cohort Study
Source: PLoS Negl Trop Dis. 2019 May 28;13(5):e0007434. doi: 10.1371/journal.pntd.0007434 (PMC6555536; doi:10.1371/journal.pntd.0007434)
Supplement: S2 Table — (DOCX) [file pntd.0007434.s002.docx]

**S2 Table. The characteristics and clinical manifestations of the SFTS patients with or without diabetes.**

| **Characteristic** | | **Diabetes** | | | |
| --- | --- | --- | --- | --- | --- |
|  |  | **Yes (n=142)** | **No (n=1954)** | **P value** | **Adjust**  **P value^#^** |
| **Demographic characteristics** |  |  |  |  |  |
| Male gender/ No. (%) | | 43 (30.3) | 814 (41.7) | 0.008 ^a^* |  |
| Age, years, mean±SD | | 62.9±9.2 | 61.3±12.4 | 0.135 ^b^ |  |
| Time from disease onset to admission, days, median (IQR) | | 6 (4-7) | 5 (4-7) | 0.038 ^c^* |  |
| **Clinical manifestations** | |  |  |  |  |
| Fever | | 142 (100) | 1954 (100) | NA | NA |
| Dizziness | | 26 (18.3) | 403 (20.6) | 0.509 ^a^ | 0.520 |
| Headache | | 13 (9.2) | 265 (13.6) | 0.135 ^a^ | 0.138 |
| Chills | | 12 (8.5) | 241 (12.3) | 0.170 ^a^ | 0.180 |
| Myalgias | | 118 (83.1) | 1606 (82.2) | 0.784 ^a^ | 0.800 |
| Lymphadenopathy | | 81 (57.0) | 1050 (53.7) | 0.445 ^a^ | 0.484 |
| Gastrointestinal symptoms | | 134 (94.4) | 1840 (94.2) | 0.922 ^a^ | 0.970 |
| Diarrhoea | | 29 (20.4) | 571 (29.2) | 0.025 ^a^* | 0.019* |
| Abdominal pain | | 7 (4.9) | 136 (7.0) | 0.354 ^a^ | 0.326 |
| Vomiting | | 46 (32.4) | 714 (36.5) | 0.321 ^a^ | 0.206 |
| Nausea | | 101 (71.1) | 1403 (71.8) | 0.863 ^a^ | 0.706 |
| Anorexia | | 114 (80.3) | 1512 (77.4) | 0.423 ^a^ | 0.532 |
| Respiratory symptoms | | 92 (64.8) | 1032 (52.8) | 0.006 ^a^* | 0.015* |
| Dyspnoea | | 22 (15.5) | 165 (8.4) | 0.004 ^a^* | 0.034* |
| Sputum | | 72 (50.7) | 762 (39.0) | 0.006 ^a^* | 0.013* |
| Cough | | 86 (60.6) | 987 (50.5) | 0.021 ^a^* | 0.039* |
| Neurological symptoms | | 65 (45.8) | 487 (24.9) | <0.001^a^ | <0.001 |
| Coma | | 30 (21.1) | 135 (6.9) | <0.001^a^ | <0.001 |
| Lethargy | | 16 (11.3) | 92 (4.7) | 0.001^a^* | 0.008* |
| Confusion | | 42 (29.6) | 317 (16.2) | <0.001^a^ | 0.002* |
| Dysphoria | | 37 (26.1) | 254 (13.0) | <0.001^a^ | 0.001* |
| Convulsion | | 39 (27.5) | 272 (13.9) | <0.001^a^ | <0.001 |
| Haemorrhagic symptoms | | 60 (42.3) | 675 (34.5) | 0.063 ^a^ | 0.251 |
| Ophthalmorrhagia | | 1 (0.7) | 11 (0.6) | 0.570 ^a^ | 0.673 |
| Ecchymosis | | 36 (25.4) | 444 (22.7) | 0.472 ^a^ | 0.794 |
| Haematemesis | | 1 (0.7) | 46 (2.4) | 0.370 ^a^ | 0.178 |
| Epistaxis | | 3 (2.1) | 13 (0.7) | 0.089 ^a^ | 0.135 |
| Melena | | 8 (5.6) | 130 (6.6) | 0.636 ^a^ | 0.421 |
| Haemoptysis | | 6 (4.2) | 71 (3.6) | 0.717 ^a^ | 0.984 |
| Gingival bleeding | | 21 (14.8) | 187 (9.6) | 0.045 ^a^* | 0.186 |
| Petechia | | 2 (1.4) | 45 (2.3) | 0.767 ^a^ | 0.366 |
| Macroscopic haematuria | | 2 (1.4) | 5 (0.3) | 0.076 ^a^ | 0.033* |

Note: Data are No.(%) of patients, mean±standard deviation, or median (IQR).

^a^ By means of the χ^2^ test or Fisher exact test

^b^ By means of the t test.

^c^ By means of the nonparametric test.

*P < 0.05

^#^ Adjusting for age, sex, time from disease onset to admission and treatment regimens (ribavirin, corticosteroid and immunoglobulin) by applying logistic regression model.
