# Supplementary material for: Preexisting chronic conditions for fatal outcome among SFTS patients: An observational Cohort Study
Source: PLoS Negl Trop Dis. 2019 May 28;13(5):e0007434. doi: 10.1371/journal.pntd.0007434 (PMC6555536; doi:10.1371/journal.pntd.0007434)
Supplement: S3 Table — (DOCX) [file pntd.0007434.s003.docx]

**S3 Table. The characteristics of SFTS patients with three levels of maximum glucose during the whole course.**

| **Characteristic** | **Maximum glucose** | | | | |
| --- | --- | --- | --- | --- | --- |
|  | **<7.0 mmol/L**  **(n=1176)** | | **7.0-11.1 mmol/L**  **(n=521)** | **≥11.1 mmol/L**  **(n=243)** | **P**  **value** |
| **Demographic characteristics** |  |  |  |  |  |
| Male gender/ No. (%) | 510 (43.4) | | 194 (37.2) | 85 (35.0) | 0.009^a^* |
| Age, years, mean±SD | 60.4±12.9 | | 63.3±10.7 | 64.0±9.8 | <0.001^b^ |
| Time from disease onset to admission, days, median (IQR) | 5 (4-6) | | 5 (4-7) | 6 (5-7) | <0.001^c^ |

Note: Data are No.(%) of patients, mean±standard deviation, or median (IQR)

^a^ By means of the χ^2^ test.

^b^ By means of the analysis of variance (ANOVA).

^c^ By means of Kruskal-Wallis test.

*P < 0.05
