# Supplementary material for: Preexisting chronic conditions for fatal outcome among SFTS patients: An observational Cohort Study
Source: PLoS Negl Trop Dis. 2019 May 28;13(5):e0007434. doi: 10.1371/journal.pntd.0007434 (PMC6555536; doi:10.1371/journal.pntd.0007434)
Supplement: S4 Table — (DOCX) [file pntd.0007434.s004.docx]

**S4 Table. The characteristics of DM-SFTS patients stratified by insulin use frequency.**

| **Characteristic** | | **Insulin** | | | |
| --- | --- | --- | --- | --- | --- |
|  |  | **≤4T**  **No (n=64)** | **>4T**  **No (n=78)** | **P value** |  |
| **Demographic characteristics** |  | | | | |
| Male gender/ No. (%) | | 18 (28.1) | 25 (32.1) | 0.612 ^a^ |  |
| Age, years, mean±SD | | 65.0±8.5 | 61.2±9.5 | 0.015 ^b^* |  |
| Time from disease onset to admission, days, median (IQR) | | 6 (4-7) | 5 (4-7) | 0.539 ^c^ |  |

Note: Data are No.(%) of patients, mean±standard deviation, or median (IQR)

^a^ By means of the χ^2^ test.

^b^ By means of the t test.

^c^ By means of the nonparametric test.

*P < 0.05
