# Supplementary material for: Preexisting chronic conditions for fatal outcome among SFTS patients: An observational Cohort Study
Source: PLoS Negl Trop Dis. 2019 May 28;13(5):e0007434. doi: 10.1371/journal.pntd.0007434 (PMC6555536; doi:10.1371/journal.pntd.0007434)
Supplement: S5 Table — (DOCX) [file pntd.0007434.s005.docx]

**S5 Table. The characteristics of SFTS patients who were tested for adhesion factors on admission (stratified by the glucose on admission).**

| **Characteristic** | | **Glucose** | | | |
| --- | --- | --- | --- | --- | --- |
|  |  | **<7 mmol/L**  **No (n=89)** | **≥7 mmol/L**  **No (n=50)** | **P value** |  |
| **Demographic characteristics** |  | | | | |
| Male gender/ No. (%) | | 40 (44.9) | 13 (26.0) | 0.027 ^a^* |  |
| Age, years, mean±SD | | 61.6±11.2 | 62.8±9.7 | 0.506 ^b^ |  |
| Time from disease onset to admission, days, median (IQR) | | 6 (5-7) | 7 (5-8) | 0.243 ^c^ |  |

Note: Data are No.(%) of patients, mean±standard deviation, or median (IQR).

^a^ By means of the χ^2^ test.

^b^ By means of the t test.

^c^ By means of the nonparametric test.

*P < 0.05
