# Supplementary material for: Preexisting chronic conditions for fatal outcome among SFTS patients: An observational Cohort Study
Source: PLoS Negl Trop Dis. 2019 May 28;13(5):e0007434. doi: 10.1371/journal.pntd.0007434 (PMC6555536; doi:10.1371/journal.pntd.0007434)
Supplement: S6 Table — (DOCX) [file pntd.0007434.s006.docx]

**S6 Table. The characteristics of SFTS patients who were tested for cytokines on admission (stratified by the glucose on admission).**

| **Characteristic** | | **Glucose** | | | | | | |  |
| --- | --- | --- | --- | --- | --- | --- | --- | --- | --- |
|  |  | **<7 mmol/L**  **No (n=47)** | | **≥7 mmol/L**  **No (n=17)** | | **P value** | |  |  |
| **Demographic characteristics** |  | | | | | | | |  |
| Male gender/ No. (%) | | | 24 (51.1) | | 6 (35.3) | | 0.264 ^a^ | | |
| Age, years, mean±SD | | | 58.3±14.0 | | 62.4±13.3 | | 0.296 ^b^ | | |
| Time from disease onset to admission, days, median (IQR) | | | 5 (4-6) | | 6 (4-7) | | 0.226 ^c^ | | |

Note: Data are No.(%) of patients, mean±standard deviation, or median (IQR).

^a^ By means of the χ^2^ test or Fisher exact test.

^b^ By means of the t test.

^c^ By means of the nonparametric test.

*P < 0.05
