# Supplementary material for: Preexisting chronic conditions for fatal outcome among SFTS patients: An observational Cohort Study
Source: PLoS Negl Trop Dis. 2019 May 28;13(5):e0007434. doi: 10.1371/journal.pntd.0007434 (PMC6555536; doi:10.1371/journal.pntd.0007434)
Supplement: S7 Table — (DOCX) [file pntd.0007434.s007.docx]

**S7 Table. The characteristics and clinical manifestations of SFTS patients with or without CVH.**

| **Characteristic** | **Chronic viral hepatitis** | | | | |
| --- | --- | --- | --- | --- | --- |
|  | **Yes (n=195)** | | **No (n=1901)** | **P value** | **Adjusted**  **P value^#^** |
| **Demographic characteristics** | |  |  |  |  |
| Male gender/ No. (%) | 75 (38.5) | | 782 (41.1) | 0.469 ^a^ |  |
| Age, years, mean±SD | 61.6±10.5 | | 61.4±12.4 | 0.780 ^b^ |  |
| Time from disease onset to admission, days, median (IQR) | 5 (4-7) | | 5 (4-7) | 0.757 ^c^ |  |
| **Clinical manifestations** |  | |  |  |  |
| Fever | 195 (100) | | 1901 (100) | NA | NA |
| Dizziness | 50 (25.6) | | 379 (19.9) | 0.060 ^a^ | 0.047* |
| Headache | 32 (16.4) | | 246 (12.9) | 0.174 ^a^ | 0.202 |
| Chills | 25 (12.8) | | 228 (12.0) | 0.736 ^a^ | 0.564 |
| Myalgias | 160 (82.1) | | 1564 (82.3) | 0.939 ^a^ | 0.933 |
| Lymphadenopathy | 94 (48.2) | | 1037 (54.6) | 0.090 ^a^ | 0.087 |
| Gastrointestinal symptoms | 187 (95.9) | | 1787 (94.0) | 0.282 ^a^ | 0.377 |
| Diarrhoea | 49 (25.1) | | 551 (29.0) | 0.257 ^a^ | 0.184 |
| Abdominal pain | 15 (7.7) | | 128 (6.7) | 0.613 ^a^ | 0.675 |
| Vomiting | 81 (41.5) | | 679 (35.7) | 0.107 ^a^ | 0.116 |
| Nausea | 146 (74.9) | | 1358 (71.4) | 0.310 ^a^ | 0.296 |
| Anorexia | 157 (80.5) | | 1469 (77.3) | 0.302 ^a^ | 0.445 |
| Respiratory symptoms | 105 (53.9) | | 1019 (53.6) | 0.948 ^a^ | 0.769 |
| Dyspnoea | 16 (8.2) | | 171 (9.0) | 0.712 ^a^ | 0.628 |
| Sputum | 81 (41.5) | | 753 (39.6) | 0.600 ^a^ | 0.826 |
| Cough | 102 (52.3) | | 971 (51.1) | 0.744 ^a^ | 0.960 |
| Neurological symptoms | 65 (33.3) | | 487 (25.6) | 0.020 ^a^* | 0.045* |
| Coma | 23 (11.8) | | 142 (7.5) | 0.033 ^a^* | 0.054 |
| Lethargy | 11 (5.6) | | 97 (5.1) | 0.746 ^a^ | 0.772 |
| Confusion | 43 (22.1) | | 316 (16.6) | 0.055 ^a^ | 0.078 |
| Dysphoria | 35 (18.0) | | 256 (13.5) | 0.085 ^a^ | 0.162 |
| Convulsion | 37 (19.0) | | 274 (14.4) | 0.088 ^a^ | 0.135 |
| Haemorrhagic symptoms | 88 (45.1) | | 647 (34.0) | 0.002 ^a^* | 0.017* |
| Ophthalmorrhagia | 1 (0.5) | | 11 (0.6) | 1.000 ^a^ | 0.999 |
| Ecchymosis | 60 (30.8) | | 420 (22.1) | 0.006 ^a^* | 0.022* |
| Haematemesis | 6 (3.1) | | 41 (2.2) | 0.409 ^a^ | 0.623 |
| Epistaxis | 1 (0.5) | | 15 (0.8) | 1.000 ^a^ | 0.559 |
| Melena | 14 (7.2) | | 124 (6.5) | 0.725 ^a^ | 0.967 |
| Haemoptysis | 8 (4.1) | | 69 (3.6) | 0.738 ^a^ | 0.907 |
| Gingival bleeding | 32 (16.4) | | 176 (9.3) | 0.001 ^a^* | 0.011* |
| Petechia | 6 (3.1) | | 41 (2.2) | 0.409 ^a^ | 0.657 |
| Macroscopic haematuria | 0 | | 7 (0.4) | NA | NA |

Note: Data are No.(%) of patients, mean±standard deviation, or median (IQR).

^a^ By means of the χ^2^ test or Fisher exact test

^b^ By means of the t test.

^c^ By means of the nonparametric test.

*P < 0.05

^#^ Adjusted by age, sex, time from disease onset to admission and treatment regimens (ribavirin, corticosteroid and immunoglobulin) using logistic regression model.
