# Supplementary material for: Preexisting chronic conditions for fatal outcome among SFTS patients: An observational Cohort Study
Source: PLoS Negl Trop Dis. 2019 May 28;13(5):e0007434. doi: 10.1371/journal.pntd.0007434 (PMC6555536; doi:10.1371/journal.pntd.0007434)
Supplement: S8 Table — (DOCX) [file pntd.0007434.s008.docx]

**S8 Table. The characteristics of SFTS patients who were tested blood coagulation function on admission (stratified by CVH).**

| **Characteristic** | | **CVH** | | | |
| --- | --- | --- | --- | --- | --- |
|  |  | **Yes**  **No (n=98)** | **No**  **No (n=867)** | **P value** |  |
| **Demographic characteristics** |  | | | | |
| Male gender/ No. (%) | | 37 (37.8) | 373 (43.0) | 0.317 ^a^ |  |
| Age, years, mean±SD | | 62.7±10.5 | 62.5±11.9 | 0.850 ^b^ |  |
| Time from disease onset to admission, days, median (IQR) | | 5 (4-7) | 5 (4-7) | 0.522 ^c^ |  |

Note: Data are No.(%) of patients, mean±standard deviation, or median (IQR).

^a^ By means of the χ^2^ test.

^b^ By means of the t test.

^c^ By means of the nonparametric test.

*P < 0.05
